# Atopic Dermatitis is More than Skin Deep: A Commentary on Atopic Dermatitis and Review of Pertinent Publications from *Children* 2019–2021

**DOI:** 10.3390/children9060850

**Published:** 2022-06-08

**Authors:** Russell J. Hopp

**Affiliations:** 1 Department of Pediatrics, University of Nebraska Medical Center, Omaha, NE 68198, USA; rhopp@childrensomaha.org; 2 Children’s Hospital and Medical Center, Omaha, NE 68114, USA

The journal *Children* has a significant publication record on the topic of Atopic Dermatitis (AD) the past four years. In this commentary, the author highlights those specific articles and provides clinical pearl(s)/perspectives taken from the review of the article. The journal *Children* has included an excellent group of manuscripts and reviews of AD, and future reviews can include more recent and potential therapies. The biggest obstacle in pediatric AD care is the slow expansion (approval) of approved medication/therapies for the younger pediatric AD population, especially biologics. The author provides perspective on each manuscript and thoughts on the status of AD therapy in children.

## 1. Year 2019

*New Cosmetic Formulation for the Treatment of Mild to Moderate Infantile Atopic Dermatitis. de Lcas R et al.* [1].

Comment: There is probably nothing more vexing for caregivers and healthcare providers than picking a moisturizer for base therapy in AD children. The authors used an extensive subjective and objective data collection to monitor the benefits for a new complex topical agent. Less relevant to their product is a good review of the underlying skin dysfunction in AD children and the multiple components of their benefits to their product. The product is in lotion form and attempts to repair the multitude of skin barrier issues seen in AD. The small sample size and its eventual international availability limits the usefulness of the product.

*The Role of Probiotics in Preventing Allergic Disease. Wang, H.T. et al.* [2].

Comment: In this relatively up-to-date review of probiotics in general in allergic diseases, with an emphasis on AD and food allergy, the authors report on three systematic studies of probiotics in AD with encouraging results and two studies with more contradictory data. They conclude that further studies are warranted.

*Biologic Treatment Options for Pediatric Psoriasis and Atopic Dermatitis. Cline, A. et al.* [3].

Comment: A now somewhat dated review of biologic therapies for AD.

*Pathophysiology of Atopic Dermatitis and Psoriasis: Implications for Management in Children. Chovatiya, R. and Silverberg, J.I.* [4].

Comment: A excellent and concise review of pathophysiology and therapy options for AD. Extensive references. Worthy of attention for interested parties.

*Complementary and Integrative Therapies for Childhood Atopic Dermatitis. Adler-Neal, A.L. et al.* [5].

Comment: A Pub-Med approach to what is known or available on alternative therapies for pediatric AD. A very extensive list of probiotics used in AD is presented. A much shorter discussion on vitamins, minerals, and herbal products is presented. Interestingly, no mention of Vitamin D was included. References are limited to searched publications.

*Topical and Oral Therapies for Childhood Atopic Dermatitis and Plaque Psoriasis. Frantz, T. et al.* [6].

Comment: Both AD and psoriasis are intermingled in the review. It provides a good companion to the review of Chovatiya and Silverberg [4]. There is no discussion of in-trial topical formulations nor of biologics.

*Quality of Life and Disease Impact of Atopic Dermatitis and Psoriasis on Children and Their Families. Na, C.H. et al.* [7].

Comment: There are few greater complexities as a Pediatric Allergist than to explain the complex pathophysiology of asthma and its effect on pulmonary function, especially when severe asthma is present, as patients may facilitate to their internal disease. Not as difficult is AD, when the disease’s consequences are so visible. The indices of Quality of Life (QoL) scales in AD are discussed. Good references are provided.

## 2. Year 2020

*The Role of the Microbiome in Food Allergy: A Review. Nance, C.L. et al.* [8].

Comment: The skin in patients with atopic dermatitis (AD) has a significant skewing to an abnormal microbiological population. It is conceivable that a GI bacteria or skin-applied bacterial transplant could have a future role(s) in AD therapy or earlier resolution.

## 3. Year 2021

*Breastfeeding and Allergic Disease: What’s New? Nuzzi, G. et al.* [9].

Comment: The role of breast feeding as a protection from AD is limited. In some circumstances, partially digested food transferring from the breast milk to the child may be a cause for sensitization or potentially triggering atopic dermatitis flares in infants, and this topic has been reviewed recently [10].

*Addressing Common Misconceptions in Food Allergy: A Review. Anagnostou, A.* [11].

Comment: Sensitization to food is not uncommon in AD in children. Food allergy becomes a true IgE-mediated disease with immediate and potentially significant outcomes. The astute clinician needs to separate sensitization from AD causation but also be aware of concomitant sensitization and food allergy in an AD patient.

*Atopic Manifestations in Children Born Preterm: A Long-Term Observational Study. Pagano, F. et al.* [12].

Comment: In a case–control study, gestational diabetes was significantly associated with AD, and pre-term birth before 29 weeks was negatively associated with AD. The relatively small numbers from a single neonatal center may not hold up in large populations, but extensive references provide some perspective.

*The Association between the Concentration of Heavy Metals in the Indoor Atmosphere and Atopic Dermatitis Symptoms in Children Aged between 4 and 13 Years: A pilot study. Choi, H.S. et al.* [13].

Comment: A selected population in cities on a Korean island served as the study basis. Indoor pollutants and heavy metals were measured in homes of children with or without AD. Significant differences for children’s AD were found in volatile organic compounds (VOC) and lead in their homes (elevated for both). The small sample size limits the applicability.

*Skin Disease in Children: Effects on Quality of Life, Stigmatization, Bullying, and Suicide Risk in Pediatric Acne, Atopic Dermatitis, and Psoriasis Patients. Kelly, K.A. et al.* [14].

Comment: Using a Pub-Med search strategy, quality of life, bullying, and suicide risk were explored in several chronic skin conditions, including AD. These issues are important but often overlooked downsides to the lives of children with AD. They also explored the potential that allergic inflammation has CNS effects.

*Barrier Impairment and Type 2 Inflammation in Allergic Diseases: The Pediatric Perspective. Ghezzi, et al.* [15].

Comment: Although not directly about AD, this review discussed an under-recognized component of AD: the barrier defect that maybe intrinsic to AD but certainly becomes worse with AD. This is a review worthy of mention for its background review of the barrier dysfunction in AD but also asthma and eosinophilic esophagitis. A worthy read.

## 4. Comments

Atopic dermatitis, along with asthma and eosinophilic esophagitis are exceedingly complex allergic diseases. A small consolation is the greater potential for resolution of AD during the pediatric years [16], although its presence during early childhood can be a great concern for parents and medical providers. A recent review discusses the role of pre-birth and early life development of food allergy and its frequent companion, atopic dermatitis, and provides a perspective for understanding the process to its onset [10].

The prevention of atopic dermatitis is an incomplete picture, with parental atopic dermatitis having a strong genetic predilection, although the co-factor of filaggrin mutations also has a strong association. Overcoming genetic factors allowing AD to occur may be difficult [17]. Potential options might include the optimization of Vitamin D levels in the mother and possible modifications of the maternal microbiome, possibly with dietary changes [18].

The early childhood presentation of eczematoid skin conditions, coupled with underlying filaggrin mutations presents a perplexing scenario for halting the atopic march [19]. Although controversial, the cautious avoidance of food the child is sensitized to may be of assistance [10]. The prompt improvement in the inflammatory skin component may support continues avoidance, but requires careful re-addition of the food in the future, with only limited avoidance, unless of overwhelmingly conclusive benefit.

Early moisturizers addition has had a role as a prevention product. This approach was recently summarized [20]. It is possible that their value might be enhanced in infants with significant aberration in barrier proteins (filaggrin), although such an approach is currently lacking.

Other pre-AD development strategies, in part, could include vitamin D supplementation, pro-biotics, or nutritional modifications. Whether aggressive microbiome adjustment in highly susceptible children might be of future value has been postulated [21]. Bleach-bath therapy has had popularity and is a reasonable regimen when effective [22].

At present, biological modifiers are not approved in the United States for atopic dermatitis until age 6. Duplimab, an anti-IL-/IL-13 monoclonal antibody, is, however, in clinical trial in children as young as 6 months, with preliminary data presented at the Revolutionizing Atopic Dermatitis Conference in 2021 [23]. Future considerations after its release included duration of therapy, stretch dosing, and relapse rates after stopping. The underpinning of these strategies is the known natural history of pediatric eczema, [24] although severe pediatric AD has a less optimistic future in long-term studies [25].

Consider the pre-biologic medication sphere (specialty) physicians live in when taking care of moderate to severe pediatric AD patients [26]. Topical steroids are effective but need to be continually/regularly used for maintenance, a message that many primary care providers and parents are hesitant to comply with. Non-steroidal topical products are often less effective as stand-alone therapy, are more expensive, are limited by age, are not generic in many situations, and are often formulary restricted and/or require prior authorization. Finally, methotrexate and cyclosporin have been long-standing therapies for severe AD children, but each have major difficulties in administration and/or potential side effects. Until a biologic is released for all pediatric age groups, this is the world pediatric AD patients live in, which often results in major frustrations for patients, parents, and care providers.

## 5. Conclusions

With a current 2020 impact factor of 2.863, the past four years has seen an impressive group of manuscripts in *Children*, particularly Reviews on chronic skin conditions in children and especially atopic dermatitis. Topics have ranged from basic pathophysiology to treatment to psychological components of chronic skin disease. This has been an outstanding grouping of articles, with the potential to add to the care of children with AD. However, major limitations persist in AD prevention and early childhood therapy. With new therapies forthcoming, additional publications in *Children* on the atopic dermatitis care of children and adolescents are welcomed.
